# The geography of smallpox in England before vaccination: A conundrum resolved

**DOI:** 10.1016/j.socscimed.2018.04.019

**Published:** 2018-06

**Authors:** Romola Jane Davenport, Max Satchell, Leigh Matthew William Shaw-Taylor

**Affiliations:** a Cambridge Group for the History of Population and Social Structure, Department of Geography, Downing Place, Cambridge, CB2 3EA, UK; b Cambridge Group for the History of Population and Social Structure, Faculty of History, West Road, Cambridge, CB3 9EF, UK

**Keywords:** England, Smallpox, Vaccination, Variolation, Endemicisation, Disease control, Historical epidemiology

## Abstract

Smallpox is regarded as an ancient and lethal disease of humans, however very little is known about the prevalence and impact of smallpox before the advent of vaccination (c.1800). Here we use evidence from English burial records covering the period 1650–1799 to confirm a striking geography to smallpox patterns. Smallpox apparently circulated as a childhood disease in northern England and Sweden, even where population densities were low and settlement patterns dispersed. However, smallpox was a relatively rare epidemic disease in southern England outside the largest cities, despite its commercialised economy and the growing spatial interconnectedness of its settlements. We investigated a number of factors hypothesised to influence the regional circulation of smallpox, including exposure to naturally occurring orthopox viruses, settlement patterns, and deliberate preventative measures. We concluded that transmission was controlled in southern England by local practices of avoidance and mass inoculation that arose in the seventeenth and eighteenth centuries. Avoidance measures included isolation of victims in pest houses and private homes, as well as cancellation of markets and other public gatherings, and pre-dated the widespread use of inoculation. The historical pattern of smallpox in England supports phylogenetic evidence for a relatively recent origin of the variola strains that circulated in the twentieth century, and provides evidence for the efficacy of preventative strategies complementary to immunisation.

## Highlights

• Large north-south differences in smallpox patterns in England pre-vaccination.
• Smallpox was controlled by isolation and deliberate infection.
• Behavioural mechanisms were sufficient to reduce transmission without vaccination.
• Historical smallpox patterns support relatively recent emergence of modern subtypes.

## 1 Introduction

Smallpox is widely considered one of the most lethal of all human pathogens, and was also the first disease to be eradicated. Vaccination was developed by Edward Jenner at the very end of the eighteenth century (Jenner, 1798), and reduced smallpox to a relatively minor cause of death in Europe by the mid-nineteenth century. However, smallpox still accounted for some 10–15 million cases annually as late as 1967 (Fenner et al., 1988: 175), and was only finally eliminated by a very concerted global campaign that combined mass vaccination with stringent isolation, contact-tracing and targeted ring-vaccination (Fenner et al., 1988: Foege, 2011). Smallpox was officially declared eradicated in 1980, although laboratory samples are still retained.

The precocious success of vaccination means that we know relatively little about the epidemiology of smallpox in unvaccinated populations. This is of more than just historical interest, because most of the global population currently lacks immunity against smallpox. Fears regarding the deliberate release of smallpox by bioterrorists in the wake of the September 11th, 2001 attacks led to a spate of mathematical modelling of the likely consequences of such an event. These models produced widely divergent recommendations, ranging from mass vaccination of national populations, to containment via case isolation and contact tracing. A major reason for the extent of discrepancies in model outcomes was the lack of robust historical evidence regarding the spread of smallpox in unvaccinated populations, which made it difficult to estimate some of the basic parameters of epidemic models (e.g. Eichner, 2003; Gani and Leach, 2001; Meltzer et al., 2001, Appendix 1).

Smallpox is caused by the orthopox virus variola (VARV). Several strains of varying virulence have been identified, and phylogenetic analyses of historical samples indicate that the variants that circulated in Europe before the twentieth century belonged to the most lethal strain, variola major (e.g. Li et al., 2007; Duggan et al., 2016). Smallpox was transmitted person to person (and to a lesser extent via infected objects), and conferred lifelong immunity on survivors. It is therefore regarded as a classic ‘crowd disease’, dependent on relatively large populations of susceptible hosts for continued transmission. The predicted historical dynamics of such person-to-person, immunising diseases were described best by the historian William McNeill. He argued that as human populations grew and became better integrated through trade and migration then disease introduction would have become more frequent and regular, and the accumulation of hosts more rapid, sustaining regular epidemics. As the frequency of epidemics increased and populations coalesced into large unified disease pools then few individuals escaped infection in childhood and most adults would have been immune. By this point smallpox would have become an endemic childhood disease that no longer required reintroduction into the population (McNeill, 1976). McNeill also recognised that this endemicisation process could involve a transitional phase, where immunising diseases such as smallpox had become endemic childhood diseases amongst long-term urban residents, but remained relatively infrequent and epidemic in surrounding rural areas. In this situation adult migrants to towns were often immunologically naive, and fell victim to urban diseases upon arrival, producing bimodal patterns of smallpox infection by age (McNeill, 1980).

The chronology of the endemicisation of smallpox in European populations remains unclear, because we have few records of causes of death before the nineteenth century. Although smallpox is usually described as an ancient disease of humans, both historical and molecular phylogenetic evidence generally supports a relatively recent origin of the variola major strain that circulated between the seventeenth and twentieth centuries (Carmichael and Silverstein, 1987; Duggan et al., 2016; Duncan et al., 2005). In London, by 1700 Europe's largest city, smallpox increased from 4 to 6% of all burials in the mid-seventeenth century to over 10% in the third quarter of the eighteenth century, and the frequency of epidemics increased from roughly four yearly to a biennial cycle over the same period (Davenport et al., 2016; Duncan et al., 1996; Krylova, 2011). Amongst London Quakers smallpox accounted for only 10% of all burials of children aged 2–4 years in the period 1650-99, but 26–29% in the period 1700-99 (Landers, 1993: 154). Thus smallpox appears to have become a more common cause of death over the course of the seventeenth and early eighteenth century in London, and may have become a more frequent epidemic disease outside the capital over the same period (Carmichael and Silverstein, 1987; Creighton, 1894: 434–445).

Where smallpox was recorded as a cause of death, then such records provide unusually rich information about the incidence of the disease. Although historical cause of death records must always be treated with great suspicion, smallpox was relatively distinctive in its symptoms and was considered by contemporaries to be confused only with chickenpox, which was rarely lethal (e.g. Buchan, 1774: 161; Creighton, 1894: 530). Estimates of case-fatality rates associated with variola major range from 10 to 30%, meaning that a sizeable proportion of all those infected can be detected in burial records where smallpox burials were noted. Smallpox was sufficiently virulent to kill adults as well as children, and was apparently relatively insensitive to host nutritional status (Dixon, 1962; Fenner et al., 1988: 196). The relatively small differences in case-fatality rates by age mean that the age structure of smallpox burials provides some indication of the age profile of those infected (Creighton, 1894: 520, 618; Nishiura et al., 2008). Because smallpox infection conferred lifelong immunity on survivors, then where adult victims were rare it is likely that most adults had already encountered smallpox in childhood. The age of smallpox victims therefore provides some indication of transmission patterns within a population.

In Sweden, where causes of death were reported for the population as a whole from 1749, smallpox accounted for 8.3% of deaths nationally in the period 1774-95 (when smallpox was first reported separately from measles) (Sköld, 1996: 549). In Finland, it accounted for 11% of deaths between 1776 and 1800 (Pitkanen et al., 1989: 98). In Sweden as a whole, 95% of smallpox deaths occurred at ages under ten years in the period 1788-92, and less than 10% of smallpox deaths were aged ten years or more in all dioceses except the very isolated island of Gotland (Sköld, 1996: 580). This pattern is consistent with the circulation of smallpox as an endemic disease of childhood.

Britain was much more densely settled than Sweden, and experienced very rapid urbanisation in the eighteenth century, with the proportion living in large towns (10,000+) rising from c.13% to over 20% in England and Wales in the course of the century (De Vries, 1984: 39). Thus, we might expect that smallpox was, as in Sweden, an endemic childhood disease and major cause of death by the mid-eighteenth century. However, the fragmentary records that exist for England present a conundrum. The few records we have that reported causes of death in the eighteenth century indicate that smallpox was a very major cause of death in some of the larger towns and cities, accounting for 10–20% of all burials in London, Manchester and a number of other northern towns (Davenport, in press). However, although smallpox was constantly present in London and was a childhood disease of urban-born residents, around 20% of smallpox burials were of young adults in eighteenth century London (Davenport et al., 2011, 2016; Landers, 1993: 153-56). Young adults constituted the main source of migrants to London, and the bimodal age pattern of smallpox victims confirms McNeill's prediction regarding the vulnerability of rural-urban migrants (McNeill, 1980). However, it also implies that London's migrant hinterland in this period included significant areas where much of the population grew to adulthood without encountering smallpox. In contrast, in mid-eighteenth-century Manchester in northern England less than 5% of smallpox burials were aged ten or over, despite the similarities in age structure of the populations of Manchester and London (Davenport et al., 2016). These differences in urban smallpox patterns appear to reflect a wider north-south divide in smallpox patterns tentatively identified by Razzell (2003). Using a small sample of burial registers that recorded smallpox burials, Razzell reported that adult smallpox victims were rare in northern England, but common in southern England. He interpreted this pattern to imply major differences in exposure to smallpox between the two regions (Razzell, 2003: x - xiv).

To investigate these intriguing patterns further we exploited the recent explosion in digital data for genealogists to search millions of burial records for mention of smallpox. Although very few parish registers consistently recorded causes of death, incumbents often noted deaths from smallpox, as well as plague and violent deaths. We identified 225 burial registers from the period 1540–1799 that reported smallpox burials and included some indication of age of victims. We then used the age patterns of smallpox victims to infer the frequency of epidemics, and tested four main theories regarding the geography of these patterns.

## 2 Methods

### 2.1 Data

To identify smallpox burials we searched c.7 million burial records donated by family history and genealogical societies in electronic form (see Table S1 for sources and geographical coverage), as well as commercial genealogical databases, using variants of the search terms ‘smallpox’ and ‘variola’. We also searched published sources of data for specific parishes identified in the secondary literature as reporting causes of death. Where smallpox burials were recorded then we evaluated the quality of recording of age information. Some registers recorded exact age in years, and some gave explicit age indicators such as ‘infant’, ‘child’, ‘young man’. The majority recorded relationships that could be used to infer adult or child status, such as ‘son of’, ‘daughter of’, ‘wife of’, ‘widow of’ (e.g. Table S2). We classified all descriptors of the types ‘son of’, ‘daughter of’ and ‘child of’ as child burials, and all marital and occupational descriptors as adult. Single women and adult males of any marital status were rarely ascribed a relationship, and their adult status was inferred either from occupational or status information (e.g. ‘labourer’, ‘gent’), or from the lack of any descriptor. This procedure could obviously create an adult bias if many entries lacked information for reasons other than age or marital status, and so only registers where more than 60% of all entries contained relationship indicators for the decades surrounding the period of smallpox recording were included, because tests indicated that where all ages were recorded then 30–50% of entries were children, which constituted a minimum threshold of expected recording of relationships. The potential for misclassification of adults as children is addressed in Supplementary Materials S1.

Adult burials were defined as aged fifteen years or older, because a number of studies of English communities have indicated that at least half of children on average left home in their mid to late teens in the seventeenth and eighteenth centuries, although this varied according to local economic opportunities (Wall, 1987). In London parishes, where both age and relationship indicators were available in burial registers, very few individuals aged twelve or more were described as ‘son’ or daughter’, reflecting the very early age at leaving home of urban residents and of in-migrants (Davenport et al., 2011). We identified 208 parishes or towns with at least ten smallpox burials and with high levels of recording of age indicators, in the period 1550–1799 (that is, from soon after the beginning of parochial registration of burials, to the initial adoption of vaccination). Where we had data for more than one parish in a town then we combined the data for these parishes to avoid double-counting settlements. Of these 208 parishes or towns, 66 additionally reported causes of death for at least 95% of entries for a minimum of ten years or more, and a further ten were identified where causes were recorded but no age information was available.

### 2.2 Hypotheses to explain the geography of smallpox endemicisation in Britain

We evaluated four hypotheses offered to explain either (1) the rarity of adult smallpox victims in northern Britain, or (2) the rarity of smallpox outbreaks in southern England. These are described and evaluated briefly below, and then tested in a regression model.

**Fig. 1:**
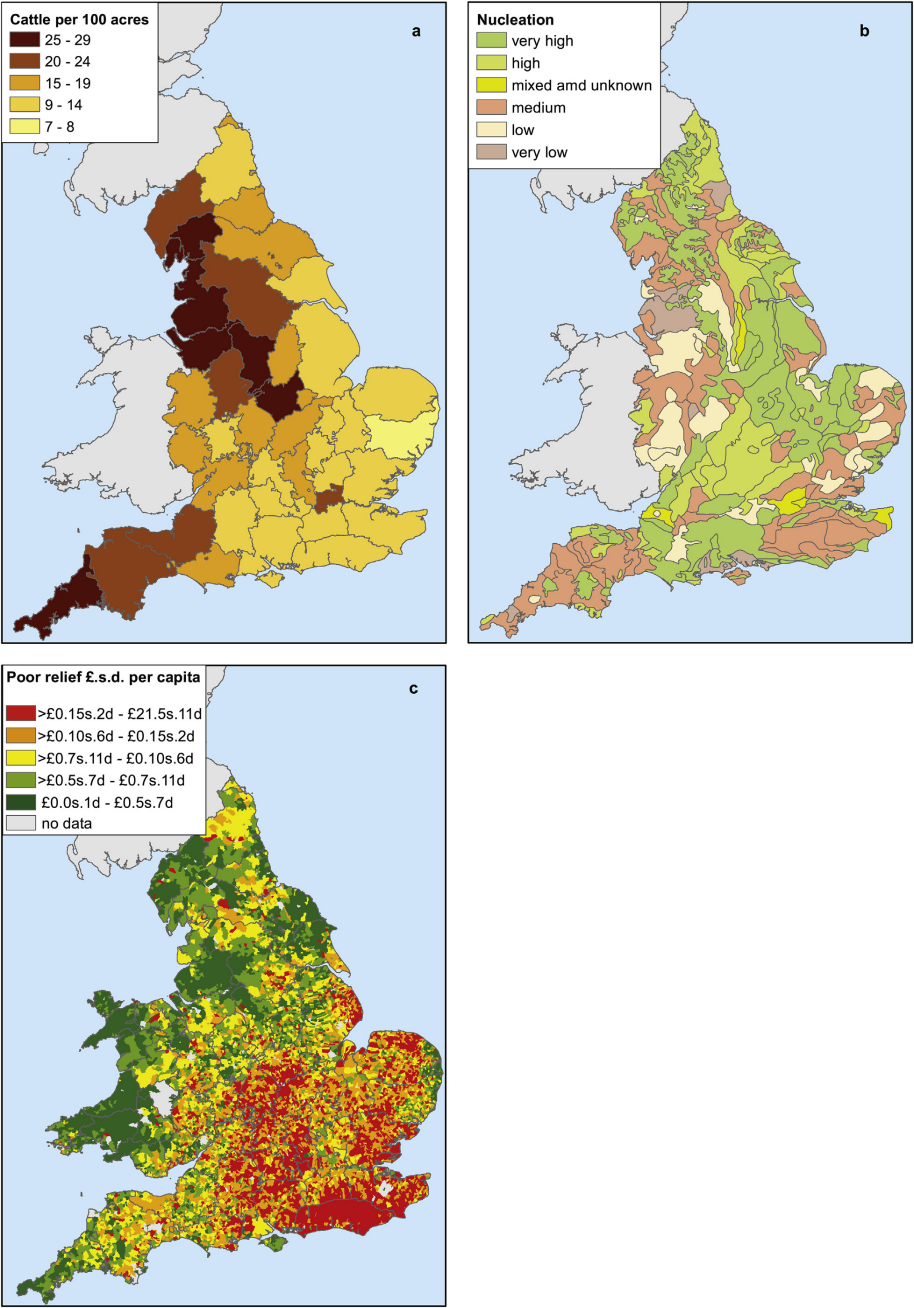
Cattle densities per 100 acres in 1870 (a), settlement nucleation patterns (b), and per capita poor law expenditure by parish in 1803 (c). Source: Parliamentary Papers, 1803-04, Parliamentary Papers, 1870; Lowerre et al. (2011).

Hypothesis 1 Infection with other orthopox viruses conferred immunity to smallpox, and the extent of such infection varied regionally (South, 2010). There is little evidence that cowpox was widely used to immunise humans against smallpox before Jenner's publication of the discovery of vaccination in 1798 (Baxby, 1981: 37). However accidental infection with natural cowpox or other orthopox viruses could have conferred widespread natural immunity to smallpox, and this phenomenon could have varied geographically. This hypothesis was difficult to test because the distribution of cowpox and related viruses in England in the eighteenth century is unknown. Even in the case of cowpox, proxies such as dairying or cattle-keeping are unavailable. However, patterns of pastoral agriculture in Britain were largely determined by soils and climate, and therefore the density of cattle reported in the 1870 Agricultural Census was a reasonable proxy for longstanding historical variations in cattle-keeping, and reflected the strong geological divide within England between north-western pastoral regions with poor soils and the arable south-east (Fig. 1a).

Hypothesis 2 Smallpox transmission was more sustained in areas of dispersed settlement (Brunton, 1992: 409; Razzell, 2003: xvi). It has been argued that infectious diseases with low infectiousness can meander through a dispersed population, leaving enough susceptible hosts to enable the pathogen to double back and sustain transmission (Cliff et al., 2000: 97–115). However in densely settled nucleated populations transmission is more successful and epidemics are explosive, leaving fewer susceptible hosts to sustain transmission between epidemics. Consistent with this hypothesis, nucleated settlement was more common in south-eastern England than in northern or south-western England. However, areas of predominantly dispersed settlement were also common in the south-east (Fig. 1b).

**Fig. 2:**
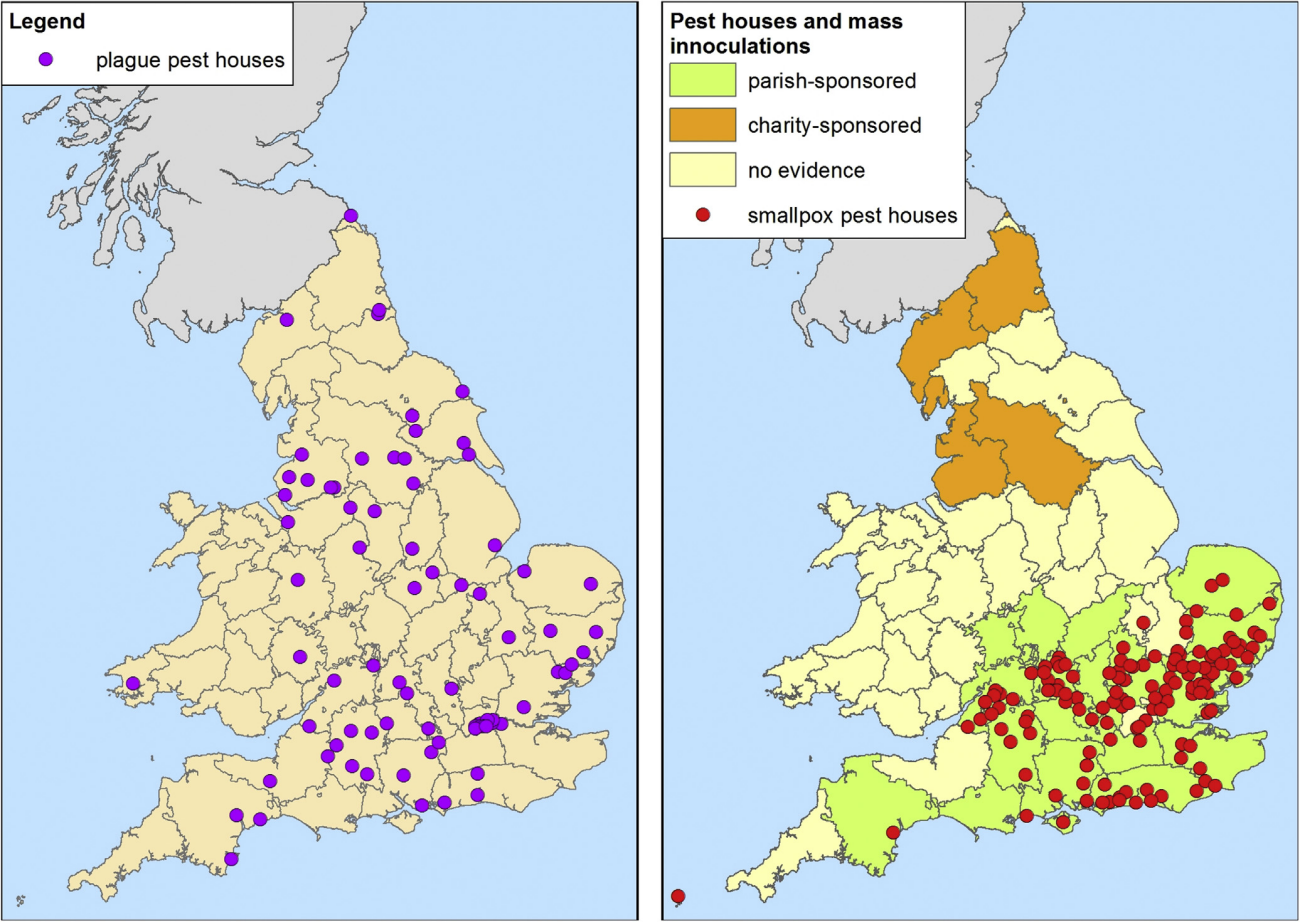
The geographical distribution of pest houses associated with plague (c.1560–1666) (a), and mass inoculations and pest houses, 1700–1799 (b). *Sources*: British History Online (including Victoria County Histories); Brunton, 1990, appendix VII; Crook (2006); Currie (2005); Dobson (1997); National Archives online catalogue; Smith, 1987; Shrewsbury, 1971; www.workhouses.org.uk.

**Fig. 3:**
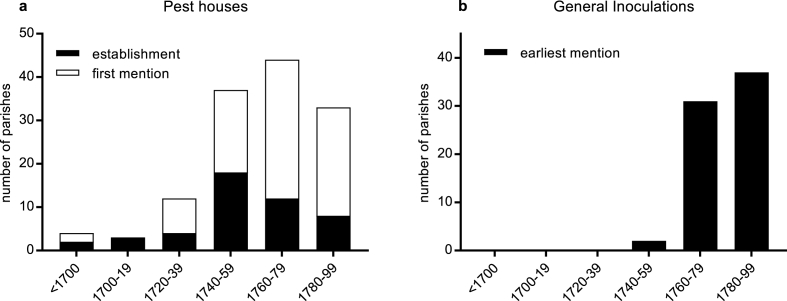
Distribution of references to pest houses in use in the eighteenth century, and General Inoculations, by earliest reference identified for each place (first mention if date of establishment not known). Sources: see Fig. 5.

Hypothesis 3 regional differences in response to smallpox resulted in effective isolation of smallpox victims in southern England and not in northern England (Haygarth, 1793: 31–35, 83, 185). Documentary sources indicate widespread attempts to avoid smallpox exposure in southern England, including isolation of infected individuals within households (Leadbeater, 2015) and closure of markets and courts when smallpox epidemics were present (e.g. Crook, 2006; Leadbeater, 2015; Razzell, 2003; Smith, 1987). No such measures have come to light in northern English sources. The most readily quantifiable evidence of smallpox avoidance is the use of pest houses to isolate (mainly poor) smallpox victims. Pest houses were widely used to isolate suspected victims of bubonic plague in the sixteenth and seventeenth centuries, across England (Fig. 2a). Most were temporary structures erected during epidemics. Literature searches for evidence of post-plague pest houses indicated that their primary use in the eighteenth century was to house smallpox victims. Of 148 pest houses confirmed as in use after 1670 (when plague disappeared from England), smallpox was mentioned as a reason for foundation in 33/38 (87%) of those where a reason for establishment was given in the source. Additionally, 44% (65/148) of all post-1670 pest houses identified were documented as used to house smallpox victims, although many of the sources gave no indication of use. Strikingly, eighteenth century pest houses were apparently confined to southern England, and this pattern contrasted strongly with the ubiquitous use of pest houses in the plague period (Fig. 2a and b). It is possible that pest houses were also in use in northern England in the eighteenth century, but were more ephemeral and therefore not detected by our method (although this was not the case with respect to seventeenth century plague pest houses). Our documentary evidence suggested that pest houses were used since the late seventeenth century to house smallpox victims, and that their use increased across the eighteenth century (Fig. 3a) (although it is probable that at least part of this apparent increase between the late seventeenth and the late eighteenth century is an artefact of increasing source abundance).

Hypothesis 4 artificial immunisation against smallpox was practiced systematically in southern but not northern England (Brunton, 1990: 148, 172). Before vaccination with cowpox (or vaccinia), other methods of artificial immunisation against smallpox were used (termed ‘variolation’ or ‘inoculation’) that involved insertion of a small amount of smallpox matter into an incision in the skin. The aim was to produce a mild case of smallpox that conferred lasting immunity. A form of the practice was introduced into England in the early 1720s, but it did not become popular until the 1760s when a cheaper and safer practice was developed. From the 1760s some southern parishes began to practice intermittent mass inoculations, with the aim of immunising all susceptible residents simultaneously (Razzell, 2003; Smith, 1987). Because inoculation carried a risk of spreading infection, only complete and simultaneous inoculation of all susceptible individuals, both adults and children, could prevent an outbreak. Such mass immunisations were only undertaken when an epidemic threatened, since they carried a risk of death from inoculation, and they were also expensive. The parish, the local unit of taxation for welfare purposes, paid for inoculation of the poor in most cases. Counties containing parishes where mass inoculations are documented to have occurred are plotted in Fig. 5b, and confirm that this practice was an exclusively southern phenomenon. In northern England partial inoculations were conducted in some of the larger towns. In these cases, free inoculation was offered but no attempt was made to achieve blanket coverage, since this would have been impossible and also pointless in large towns where smallpox was endemic. Moreover, partial inoculations in northern England were paid for by charitable individuals or institutions, not the parish (Brunton, 1990, ch. 5; May 1997). Brunton speculated that mass parish-sponsored inoculation was only viable in the relative wealthy southern arable parishes (Brunton, 1990: 149, see Fig. 1c). The timing and geography of the development of mass inoculation suggests that it was only adopted where smallpox was already a rare epidemic disease affecting adults (Fig. 2, Fig. 3b).

### 2.3 Regression modelling

To test whether explanatory variables proposed in the literature were associated with childhood smallpox (that is, low proportions of adult smallpox burials), we used multi-level regression, a form of modelling that takes into account variance at the level of individual observations and at higher levels into which individual observations are grouped, and makes it possible to compare the importance of explanatory variables that related to individual places with explanatory variables that described characteristics of the wider environment in which the settlements were situated (see below) (Steele, 2009).

Our outcome variable was the proportion of smallpox burials that were children (aged under 15). The distribution of outcome values was highly non-normal and so we constructed a binary variable: whether children aged under 15 comprised 80% or more of smallpox burials. The cut-off for the construction of the binary variable (80%) captured most of the strong geographical patterning in the data but also retained some of the within-region variation (see Fig. 5). A continuous version of the full model produced very similar results. We treated our sample as clustered at the level of county, because strong geographical patterning was evident at the county level, and because some of our explanatory variables were only available at the county level. The (random-intercept) logistic model we used took the form

ln(Y/(1-Y)) = β0 + β1x1_ij_ + β2x2_j_ + u_j_ + e_ij_

where Y was the probability that the outcome variable (the odds that child burials comprised 80–100% of smallpox burials) was equal to 1, and ×1 and ×2 were explanatory variables operating at either the level of individual settlements (*i*, for example per capita poor law expenditure), or at a higher (county) level (*j*, for example the presence of pest houses). The exponential functions of the co-efficients β1 and β2 were odds ratios (OR), such that an OR of 1.00 meant that the explanatory variable did not influence the outcome, values of OR>1 were associated with a higher odds of the outcome, and OR<1 with lower odds. The error term u_j_ captured the residual variance due to differences between counties, and e_ij_ referred to the additional variance at the level of the settlement, which was determined by the logistic distribution (Steele, 2009).

Settlements associated with burial registers in our sample were geo-referenced to the point defined for each place in the Ordinance Survey 50k Gazetteer (2013). Distances to turnpike roads, navigable waterways and ports were calculated in ArcMap 10.4.1 using shortest straight line distance from each transport feature, for single year time slices of the transport networks, extracted from a dynamic GIS (Geographical Information Systems) of the historical transport network of England and Wales (https://www.campop.geog.cam.ac.uk/research/projects/transport/data).

In the null multi-level model (containing only the outcome variable) 88% of total variance in outcomes was attributable to variance between counties, compared with 12% due to variance between individual observations, justifying the use of a multi-level model (see Table 2). The model included only observations with time spans with midpoints greater than or equal to 1760 (see Fig. 5), because the dearth of northern parishes in the sample before this date produced an unbalanced sample, and the inoculation variable only referred to practices evident between c.1760 and 1800. Explanatory variables at parish (or town) level were:

(1) Natural log of population size in 1801 (the first date at which parish-level populations were available, from the first national census). For parishes that formed part of multi-parish towns then estimates of total urban population size were used instead (Bennett, 2012).
(2) Natural log of population density in 1801.
(3) Transport connectedness in 1750, specified as a binary variable (<2 km/2 km or more from a port, navigable waterway or turnpike road).
(4) Per capita expenditure on the poor in 1803.
(5) Settlement nucleation, specified as a three level variable. Settlement nucleation was based on the relative frequencies of settlement types within a given area, at the highest level of resolution available (the ‘local region’) (Lowerre et al., 2011; Roberts and Wrathmell, 2003). ’High’ included Roberts' and Wrathmell's categories low to extremely low, ‘medium’ included categories ‘medium to low’, ‘medium’ and ‘medium to high’, and ‘low’ included values high to extremely high. This was not strictly a parish-level variable, but reflected relatively local, sub-county settlement patterns.

Explanatory variables at the county level were:

(1) Cattle density per cultivable hectare, 1870.
(2) The presence of pest houses in the eighteenth century, specified as a binary variable (presence or absence).
(3) Mass inoculation practices, specified as a categorical variable (no evidence/parish-sponsored General Inoculation/charitable mass inoculation). These were exclusive categories, at the county level.

Populations and population densities were converted to natural logs to normalise their distributions. Explanatory variables that were significantly associated with smallpox age patterns in bivariate models were then tested for inclusion in a process of stepwise subtraction from a full model, and were only included in the final model if the variable coefficient was statistically significant (P < 0.05) and if the variable improved the fit of the model, as assessed by log-likelihood ratio tests. Models were fitted using the ‘melogit’ function in Stata 14.1 (StataCorp., 2015). The final model was equivalent to an ordinary logistic regression (because the county-level variables accounted for almost all the variation at county level), and tests of goodness of fit (Hosmer-Lemeshow test), collinearity and outliers were applied to the ordinary logistic model, for simplicity.

## 3 Results

### 3.1 Smallpox patterns in eighteenth century England

**Fig. 4:**
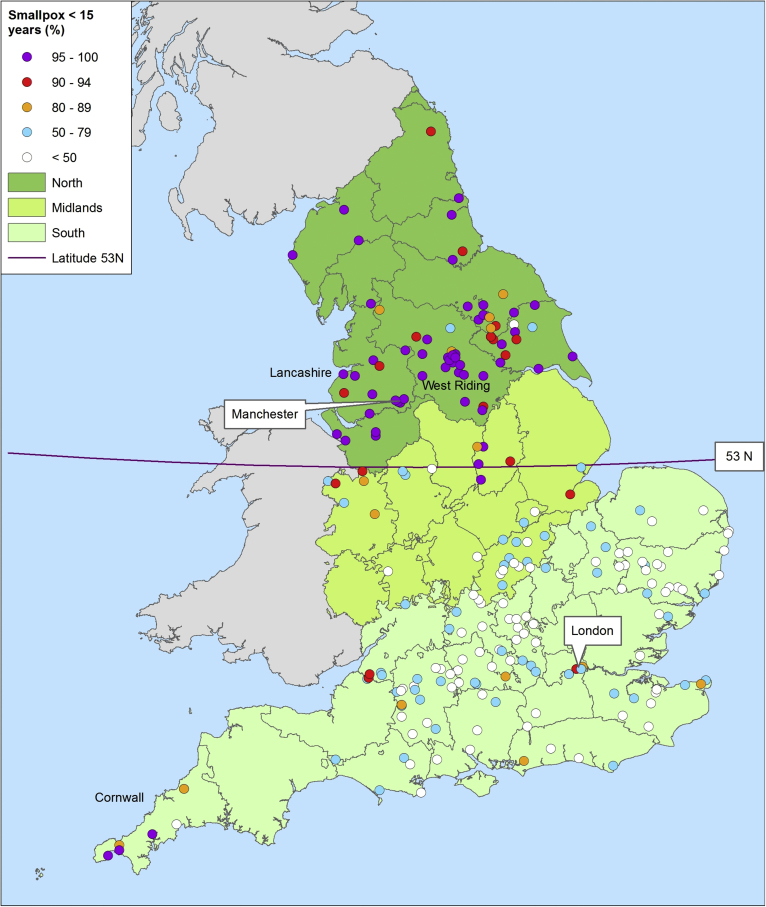
Percentage of smallpox victims aged under 15 years by place, and English regions. *Notes:* Divisions represent county borders. Sources: see Table S1.

**Fig. 5:**
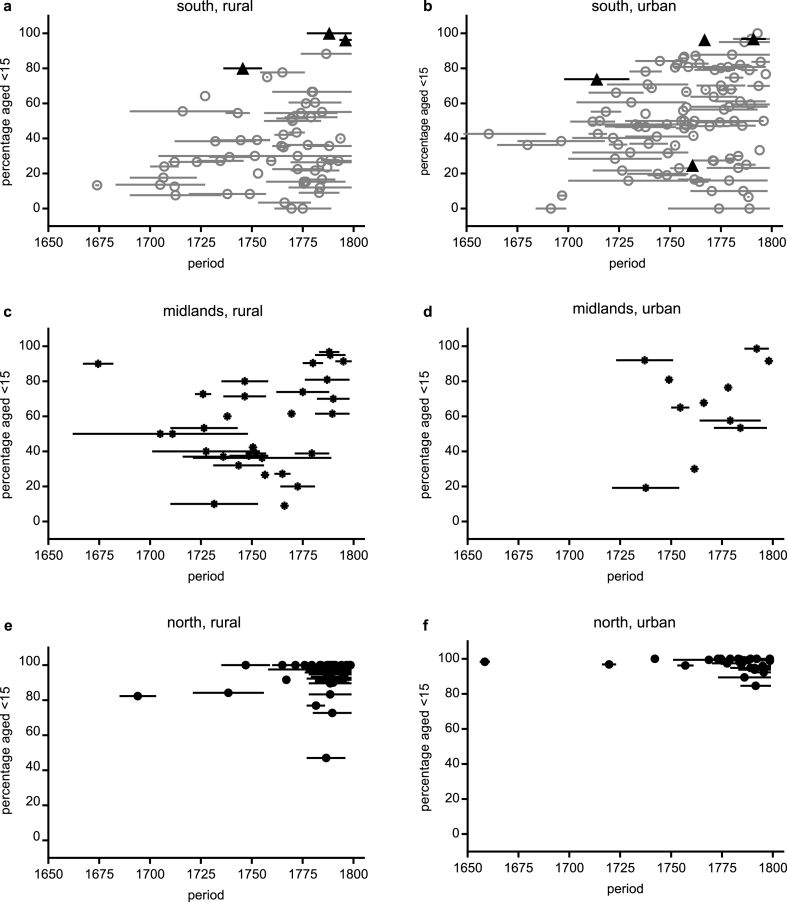
Percentage of smallpox burials aged under 15 or child by period, rural/urban and region of England. Triangles represent places in Cornwall.

The percentage of smallpox burials that were children ranged from 0 to 100% in our sample of 225 parishes and towns, and displayed a very strong geographical patterning (Fig. 4, Fig. 5). Smallpox victims were overwhelmingly children in northern England, but displayed a wide age range in southern England. The midland counties comprised an apparently transitional zone, with intermediate proportions of child victims. This pattern confirms an earlier study based on 40 parishes (Razzell, 2003).

The temporal dimension of the pattern is displayed in Fig. 5, which shows the exact percentage of child smallpox victims in each parish or town by period of observation. The sample was divided between rural and urban settlements, to compare the effects of rural-urban migration in towns. In southern England all rural settlements were characterised by a relatively low percentage of child smallpox burials (90% or less), with the exception of three rural parishes in Cornwall (Fig. 5a). Most towns in southern England were also characterised by relatively low proportions of child smallpox burials (Fig. 5b). By contrast adult smallpox victims were apparently rare in rural as well as urban settlements in northern England, relative to southern settlements, and this was apparently the case even before the mid-eighteenth century, although we had unfortunately very few records with which to test this (Fig. 5e and f). Settlements in the midlands zone displayed a wide range of values (Fig. 5c and d). The similarities between rural and urban settlements in each geographical zone were consistent with strong differences in the degree of smallpox endemicisation in each region. Before 1800 urban centres were dependent on rural immigrants for growth or even maintenance of population size, and therefore most urban populations were characterised by a bulge of young adult migrants. In this case even where smallpox was a predominantly childhood disease amongst the urban-born sector of the population, many young adults remained at risk if they had grown up in rural areas where smallpox was rare. This was clearly the case in London, and in southern English towns (Fig. 5a and b). Conversely the almost complete absence of adult smallpox victims in northern towns implied that smallpox was already a childhood disease in rural hinterlands.

**Fig. 6:**
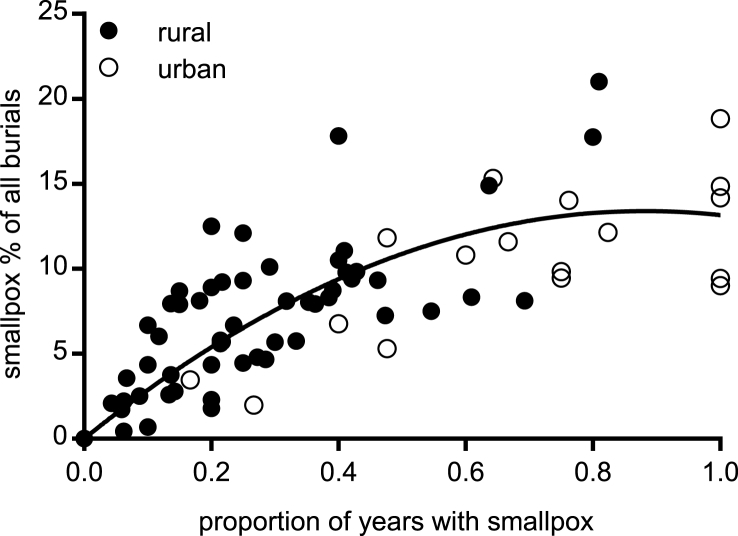
Smallpox burials percent of all burials and proportion of all years that smallpox burials were recorded. Values were fitted with a second order polynomial equation (r^2^ = 0.55, N = 72).

Where smallpox was a childhood disease then it was also a more major cause of death. We were unable to measure smallpox mortality rates directly in our sample, because we had only short runs of data with recorded smallpox burials, and because we lacked information on contemporary population sizes. To estimate the burden of smallpox we therefore measured the proportion of all burials that smallpox accounted for. For this we used a subset of 74 parishes where 95% or more of all burials were ascribed a cause of death, because where smallpox was the only cause recorded (the majority of cases) then we could not know whether the smallpox burials noted were a complete record of all smallpox deaths in a given period. In addition we used only those registers where causes of death were recorded for a minimum ten year period, to reduce the distorting effects of epidemic years. Comparing southern England (excluding Cornwall) with the rest of the country, smallpox was apparently a less important cause of death in southern England, contributing on average around 4% of all burials in rural areas in the south, compared with nearly 8% in the north (Table 1). Our sample of towns was extremely small, but the scant data we had suggested that smallpox was also a less important cause of death in southern than northern towns (Table 1), consistent with evidence that crude smallpox mortality was lower in London than it was in Manchester, despite the much larger size of London (Davenport et al., 2016). Consistent with higher smallpox mortality, the frequency of epidemics was also higher in northern England. We were unable to measure epidemic cycles directly because we had only short runs of data where all causes of death were recorded. Instead we used the number of years with smallpox burials as a proxy for epidemic frequency. Fig. 6 indicates that where smallpox was a more frequent visitor then it was also a more major cause of death (r^2^ = 0.55).

**Table 1:** Smallpox burials percent of all causes of death in burial registers where causes of death were recorded for a continuous period of 10 years or more.

region	type	smallpox % of all burials, mean	95% CI	P (south < north)^a^	N
south^b^	rural	4.08	2.37–5.78		14
north	rural	7.90	6.51–9.29	0.0004	43
south	urban	8.37	4.16–12.58		7
north	urban	13.37	10.27–16.47	0.0205	10

a P values report the results of (one-sided unpaired with unequal variance) t-tests of differences in means between north and south in each settlement type.

b The ‘southern’ sample comprised all places in dataset that were south of 53° latitude, but excluding Cornwall. The Northern sample included the rest of England.

**Table 2:** Results of multi-level logistic regression modelling of the odds that children aged under 15 years comprised 80–100% of smallpox burials.^a^

	Intercept-only model	Bivariate models	Full model
	OR (S.E.)	OR (S.E.)	Adjusted OR (S.E.)
Intercept	0.556 (0.566)		4.061 (1.797)**
Parish-level variables			
(ln) population 1801		1.818 (0.605)	
(ln) population density 1801		2.150 (0.774)*	
Distance to transport major route >=2 km		1.000	1.000
< 2 km		5.504 (5.510)	10.051 (11.085)*
(ln) Poor law expenditure, 1803		0.071 (0.067)**	
Extent of settlement nucleation: low		1.000	
medium		0.610 (0.745)	
High		0.172 (0.259)	
County-level variables			
Cattle density, 1870		1.599 (0.261)**	
pest house(s) in use 1670-1800		0.005 (0.005)***	0.016 (0.020)**
Mass inoculation: none		1.000	1.000
comprehensive, parish-sponsored		0.003 (0.004)***	0.124 (0.121)*
partial, charitable		16.523 (29.411)	6.334 (7.022)
Variance: parish-level	3.29		3.29
Variance: county-level	23.22		0.00
Deviance from intercept-only model			44.09 (4 d.f.)***
P same as ordinary logistic model	0.000		1.000

a P values P < 0.05 (*), P < 0.01 (**) P < 0.001 (***).

### 3.2 Multi-level regression modelling

Our analyses indicated that smallpox was a childhood disease in northern England, but not in southern England. This was a surprising finding because southern England, with the exception of the southwest (including Cornwall) was characterised by relatively high levels of urbanisation and population densities in the seventeenth and eighteenth centuries, factors that should have favoured the transmission and endemicisation of infectious diseases. Northern England was relatively sparsely populated especially in the seventeenth century, and characterised by poorer infrastructure (especially outside the West Riding of Yorkshire and Lancashire, indicated in Fig. 4) (see Figures S2,S3). Within our sample, there were no significant differences in parish population size or density in 1801, but the southern sample was more urban, and on average better connected to major transport routes (Table S3).

To investigate the proximate causes of the strong geography of childhood smallpox we used a multi-level regression analysis incorporating explanatory variables hypothesised to affect smallpox transmission. Results are reported in Table 2.

Our modelling exercise did not support hypotheses regarding the importance of settlement nucleation or accidental vaccination. Areas of highly nucleated settlement were not statistically associated with a high proportion of child smallpox victims in bivariate analysis. This was not surprising, because the presence of smallpox as a childhood disease showed a very strong and simple geography compared with the more complex patterns of settlement types, making it unlikely that local settlement patterns were important drivers of smallpox transmission (Fig. 1, Fig. 4). High cattle densities were strongly and positively associated with the probability of a high proportion of child smallpox victims in bivariate analysis, but this association disappeared when other variables were included in the model (Table 2). It was unlikely that pre-Jennerian vaccination could account for the lack of adult victims in northern England because overall levels of smallpox mortality appear to have been higher in northern Britain than where adult victims were more common (Table 1), whereas vaccination programmes were generally associated with falls in smallpox mortality, and rises in the mean age at death (Hardy, 1983).

The strongest predictors of childhood smallpox patterns were practices designed deliberately to stop the spread of smallpox. The presence of pest houses in a county was associated with a strong reduction in the odds that 80% or more of smallpox victims were children, holding other variables constant (OR = 0.016, P = 0.001, Table 2). The use of comprehensive mass inoculation in a county was also associated with very low odds (OR = 0.124) that children comprised 80% or more of smallpox victims (P = 0.032). However partial mass inoculations, such as those conducted in some northern towns, were associated with childhood smallpox (OR = 6.334, P = 0.096). This should not be interpreted to imply that partial inoculation promoted the spread of smallpox (indeed the causality may have run in the opposite direction). Substitution of a simple binary variable that distinguished counties with general mass inoculation from all others had very little effect on the model. The inclusion of variables for presence of pest houses and for inoculation practices accounted for most of the variance at the county level (from 87.6% of total variance in the null model to approximately 0% of unexplained variance in the full model). That is, practices designed deliberately to stop the spread of smallpox (isolation of the poor in pest houses and general immunisation) explained almost all of the strong spatial patterning observed in the sample. The final model was therefore equivalent to an ordinary logistic regression (adjusted pseudo-r^2^ of the comparable logistic model = 0.69).

Once the effects of county-level characteristics were accounted for, then the characteristics of individual parishes, such as population size and density and expenditure on the poor, were relatively unimportant in explaining variations in the percentages of smallpox victims that were children (Table 2). However being within 2 km of a major transport route (navigable waterway or turnpike road) was strongly associated with childhood smallpox (OR = 10.051, P = 0.036). This variable was not a proxy for urban settlements (substituting a dummy variable for urban places did not improve model fit and the coefficient was not significant). Rather this variable probably captured a protective effect of remoteness. Interacting this connectedness variable with northern or southern location produced similar sizes of effect of connectedness in both regions, indicating that connectedness was associated with increased likelihood of smallpox being a childhood disease regardless of region.

## 4 Discussion

The data and analyses presented here confirm previous impressionistic accounts of a strong geographical patterning of smallpox in England. Smallpox was a common endemic disease in northern Britain by the late eighteenth century, affecting mainly children even in rural communities, and accounting for 10–20% of all burials in towns. However, in southern England smallpox was apparently a relatively rare epidemic disease affecting adults as well as children and accounting for perhaps 5% or less of burials outside London. Young adults from rural areas of southern Britain were therefore at high risk of smallpox infection in towns, whereas northern migrants were not. This pattern is best explained by the partial success of deliberate strategies to inhibit smallpox transmission in southern England outside the largest cities, including the strict isolation of victims and, after 1760, artificial immunisation of whole communities. These strategies appear to have been sufficient to reduce the frequency of smallpox outbreaks and to reduce the impact of smallpox as a cause of death, compared with northern England and Sweden. This was an impressive achievement, because southern England was more densely settled and urbanised than most of northern England and Sweden in particular, and would otherwise be expected to have sustained more rapid circulation of smallpox and higher mortality. Instead it appears that a high proportion of adults survived to adulthood without accidental infection (although after 1760 many may have been protected by deliberate inoculation).

The efficacy of the preventative measures used against smallpox in eighteenth century England supports the characterisation of smallpox as a disease of low infectivity, particularly given that one pillar of prevention, mass inoculation with live smallpox, carried the risk of engendering an epidemic. The strategies deployed by southern parishes were analogous to the surveillance, isolation and ring vaccination strategies that proved vital to the success of the global eradication campaign. Unlike the global programme however the preventive measures described here were all enacted at the local level, by individual parishes. In contrast to plague epidemics, the central government issued no directives equivalent to Books of Orders with respect to smallpox control (until the nineteenth century when legislation was eventually enacted regarding vaccination). Even within areas where there is evidence that pest houses and mass immunisation were deployed, historical sources suggest that parishes varied in the extent to which they deployed these measures (Brunton, 1990: 149-50; Smith, 1987: 62, 151-2). Therefore it is likely that the strong geographical patterns of childhood and adult smallpox victims represent emergent properties of the uncoordinated actions of many parishes, with more vigilant parishes effectively creating buffer zones that protected less active parishes from smallpox transmission. The apparent efficacy of these local public health measures provides support for the importance of inclusion of behavioural changes (such as avoidance of crowded areas and other perceived infection risks) in models of smallpox outbreaks (Del Valle et al., 2005).

The communal motive to prevent smallpox outbreaks seems to have been mainly financial. Where adults were vulnerable to smallpox then documentary evidence indicates that they avoided towns during smallpox outbreaks, resulting in closure and relocation of markets, fairs and courts, and economic losses to the towns affected. Additionally, English parishes were required by law to provide for their poor members. Infected adults could require paid nursing, and if a married adult male became ill then the parish could be liable to support his family while he could not work, and to provide indefinite financial support if he died. In the case of adult women, the parish could be required to provide paid care for her children so that her husband could continue to work. Therefore where adults were at risk of smallpox then at least in southern England parishes were highly motivated to build pest houses and to pay for General inoculations. However where smallpox victims were mainly children, as in northern England, then the costs to the parish of an epidemic were small, because children were nursed by their families, rather than at parish expense, and were cheap to bury, and preventative measures were left to charitable institutions. The origins of the differences between northern and southern England in attitudes to smallpox lie beyond the scope of this paper, but may reflect basic geographical differences in the capacity of parishes to raise revenues for pay for smallpox control measures, and/or to enforce such measures (Davenport, 2018).

The preventative practices identified here do not appear to be of ancient origin, but developed over the course of the late seventeenth and eighteenth centuries. We suggest that these practices were adopted in response to the emergence of smallpox as a major public health problem in this period, as claimed by contemporaries (Creighton, 1894: 435–441). It is likely that smallpox was a rare epidemic disease throughout most of Britain in the seventeenth century, but as its incidence increased then a sufficient proportion of southern parishes responded with communal measures that prevented endemicisation, while northern parishes did not. These conclusions regarding the chronology of smallpox incidence are consistent with molecular-phylogenetic evidence for a relatively recent origin of modern smallpox strains. Analysis of smallpox DNA recovered from a seventeenth century Lithuanian mummy indicates that this smallpox strain was ancestral to all known twentieth century strains, suggesting that a new variant of smallpox emerged in the sixteenth or seventeenth century, and replaced any pre-existing variants (Duggan et al., 2016). The curious geography of smallpox in England also confirms some of the more distinctive features of the smallpox virus: its capacity to persist at relatively low host densities, and its susceptibility to relatively crude control measures. These features, together with the possibility of the late emergence of a virulent subtype, complicate McNeill's model of historical smallpox as an ancient and classic crowd disease.
