# Supplementary material for: The geography of smallpox in England before vaccination: A conundrum resolved
Source: Soc Sci Med. 2018 Jun;206:75–85. doi: 10.1016/j.socscimed.2018.04.019 (PMC5958952; doi:10.1016/j.socscimed.2018.04.019)
Supplement: Multimedia component 1 [file mmc1.docx]

**SUPPLEMENTARY MATERIALS**

**S1. Potential misclassification of adult and child smallpox burials.**

One problem with the use of relationship descriptors to infer adult or child status was that an unmarried adult child could be described in a burial register as a son or daughter if they lived with a parent. This would artificially inflate the proportion of smallpox burials that were classified as child burials, and could produce spurious geographical patterns if age at leaving home or proportions unmarried varied regionally. To test whether the age of those described as adults varied geographically we used a sub-sample of burial registers where exact age was recorded, and compared the mean age of adult smallpox victims with the percentage of adult smallpox burials as classified using our method. We considered only smallpox victims aged ten years old or more, to avoid potential biases due to under-recording of smallpox burials of young children. There was a clear positive relationship between the mean age of adult smallpox victims and the percentage of smallpox burials classified as adult (r^2^ = 0.55, Figure S1). That is, where smallpox victims were mainly children then those adults who did die from the disease were also largely in their teens or early twenties, suggesting that they comprised the tail of a young age distribution of victims. Conversely, where a high proportion of smallpox victims were adult then adult victims included many who had survived into late adulthood without encountering smallpox. Therefore geographical differences in the preponderance of adult victims of smallpox was unlikely to be an artefact of regional patterns in the recording of status or cause at burial.

**Table S1**. Sources searched for smallpox burials

| County | Source coverage | Sources |
| --- | --- | --- |
| Bedfordshire | Selected parishes (from secondary references) | Razzell (2003): xii |
| Berkshire | Most parishes | Berkshire Family History Society records; Razzell (2003): xii |
| Buckinghamshire | Most parishes | Buckinghamshire Family History Society* |
| Cambridgeshire | Most parishes | Cambridgeshire Family History Society* |
| Cheshire | Selected parishes (from secondary references) | Cheshire Archives and Local Studies Centre; Creighton (1894), vol. 2: 554 |
| Cornwall | Selected parishes (from secondary references) | Family Search original parish register images; <http://truro.homestead.com>; |
| Cumberland | Selected parishes (from secondary references) | Creighton (1894), vol. 2: 554; Razzell (2003): xi-xii |
| Derbyshire | Most parishes | Derbyshire Family History Society* |
| Devon | Most parishes | Devon Family History Society* |
| Dorset | Selected parishes (from secondary references) | Dorset Online Parish Clerks |
| Durham | Most parishes | Durham Records Online* |
| Essex | Selected parishes (from secondary references) | SEAX online catalogue; essexandsuffolksurnames.co.uk |
| Gloucestershire | Selected parishes (from secondary references) | Gloucestershire Archives |
| Hampshire | Most parishes | Hampshire Family History Society; Razzell (2003): xii; Kinstsroots.co.uk |
| Herefordshire | None identified |  |
| Hertfordshire | None identified |  |
| Kent | Most parishes | Kent Family History Society*; Medway Archives |
| Lancashire | Manchester parish and Lancashire parishes starting A-D | Lancashire Online Parish Clerks*; Manchester and Lancashire Family History Society*; this paper |
| Leicestershire | 68 parishes plus Leicester borough | Leicestershire and Rutland Family History Society |
| Lincolnshire | Selected parishes (from secondary references) | Lincolnshire Archives Linctothepast website |
| Middlesex | Selected parishes | This paper (City of Westminster Archives Centre; London Metropolitan Archives) |
| Norfolk | Most parishes | Norfolk Family History Society* |
| Northamptonshire | Most parishes | www.familyhistorynorthants.co.uk |
| Northumberland | Some parishes | Durham Records Online* |
| Nottinghamshire | Most parishes | Nottinghamshire Family History Society* |
| Oxfordshire | Selected parishes (from secondary references) | Leadbeater (2015) |
| Rutland | Most parishes | Leicestershire and Rutland Family History Society |
| Shropshire | Most parishes | Shropshire Family History Society* |
| Somerset | Some parishes | Bristol and Avon Family History Society |
| Staffordshire | Selected parishes (from secondary references) | Staffordshire Parish Register Society |
| Suffolk | Most parishes | Suffolk Family History Society* |
| Surrey | Some parishes | Surrey History Centre; West Surrey Family History Society |
| Sussex | Some parishes | www.findmypast.co.uk |
| Warwickshire | Selected parishes (from secondary references) | www.findmypast.co.uk |
| Westmorland | Some parishes | Cumbria Family History Society* |
| Wiltshire | Most parishes | Wiltshire Family History Society* |
| Worcestershire | Selected parishes (from secondary references) | Birmingham and Midland Society for Genealogy and Heraldry |
| Yorkshire | Selected parishes (with Dade-style registers or from secondary sources) | Borthwick Institute; Razzell (2003): xii; West Yorkshire Archives; Wharfedale Family History Society; Yorkshire Parish Register Society |

**Table S2**. Entries in the burial register of Lowestoft, St Margaret (Suffolk), December 1710.

| day | forename | descriptor | *age descriptor* | *age ascribed* |
| --- | --- | --- | --- | --- |
| 5 | Martha | da Thomas; smallpox | *daughter* | *child* |
| 5 | Elizabeth | Wd | *widow* | *adult* |
| 6 | James | lab; left all he had to poor abt 120s; smallpox | *labourer* | *adult* |
| 12 | William | smallpox | *none* | *adult* |
| 12 | John | chd Thomas; smallpox | *child* | *child* |
| 15 | Robert | servant John Goddle; smallpox | *servant* | *adult* |
| 16 | [daughter] | da Robert; smallpox | *daughter* | *child* |
| 16 | John | grocer; smallpox; Mr | *grocer, Mr* | *adult* |
| 19 | Mary | wi Dr Joseph | *wife* | *adult* |
| 25 | Elizabeth | da Matthew & Elizabeth | *daughter* | *child* |

**Table S3**. Population and connectness by region.^1^

|  | **Mean (95 % CI)** | | |  | |
| --- | --- | --- | --- | --- | --- |
| **Variable** | | **South** | **north** | | **P**^2^ |
| Population of rural parishes, 1801^3^ | | 814  (661 – 1,004) | 738  (523 – 1,042) | | 0.63 |
| Population of towns, 1801^3^ | | 3,099  (2,199 – 4,368) | 4,732  (2,199 – 4,368) | | 0.17 |
| Population density of parish, 1801 (persons per acre) ^3^ | | 0.37  (0.30 – 0.47) | 0.45  (0.32 – 0.63) | | 0.36 |
| Proportion of sample urban | | 0.47 | 0.31 | | 0.01 |
| Mean distance to turnpike road, waterway or port IN 1750 (km) | | 1.72  (1.29 – 2.28) | 2.23  (1.53 – 3.26) | | 0.27 |
| Mean distance to turnpike road, waterway or port IN 1800 (km) | | 1.72  (1.29 – 2.28) | 2.23  (1.53 – 3.26) | | 0.08 |
| N | | 131 | 77 | |  |

^1^ Regions defined in fn 2, Table 1.

^2^ P values report the results of (two-sided unpaired with unequal variance) t-tests of differences in means between north and south, or a two-sample test of unequal proportions (for the proportion of samples that were urban).

^3^ Populations, densities and distances were converted to natural logs to normalise their distributions. Means reported are geometric means.

**Figure S1.** Mean age of adult burials (aged ten years or more) in registers where exact age at death was recorded. Values were fitted with a second order polynomial equation (r^2^ = 0.55, N = 96).


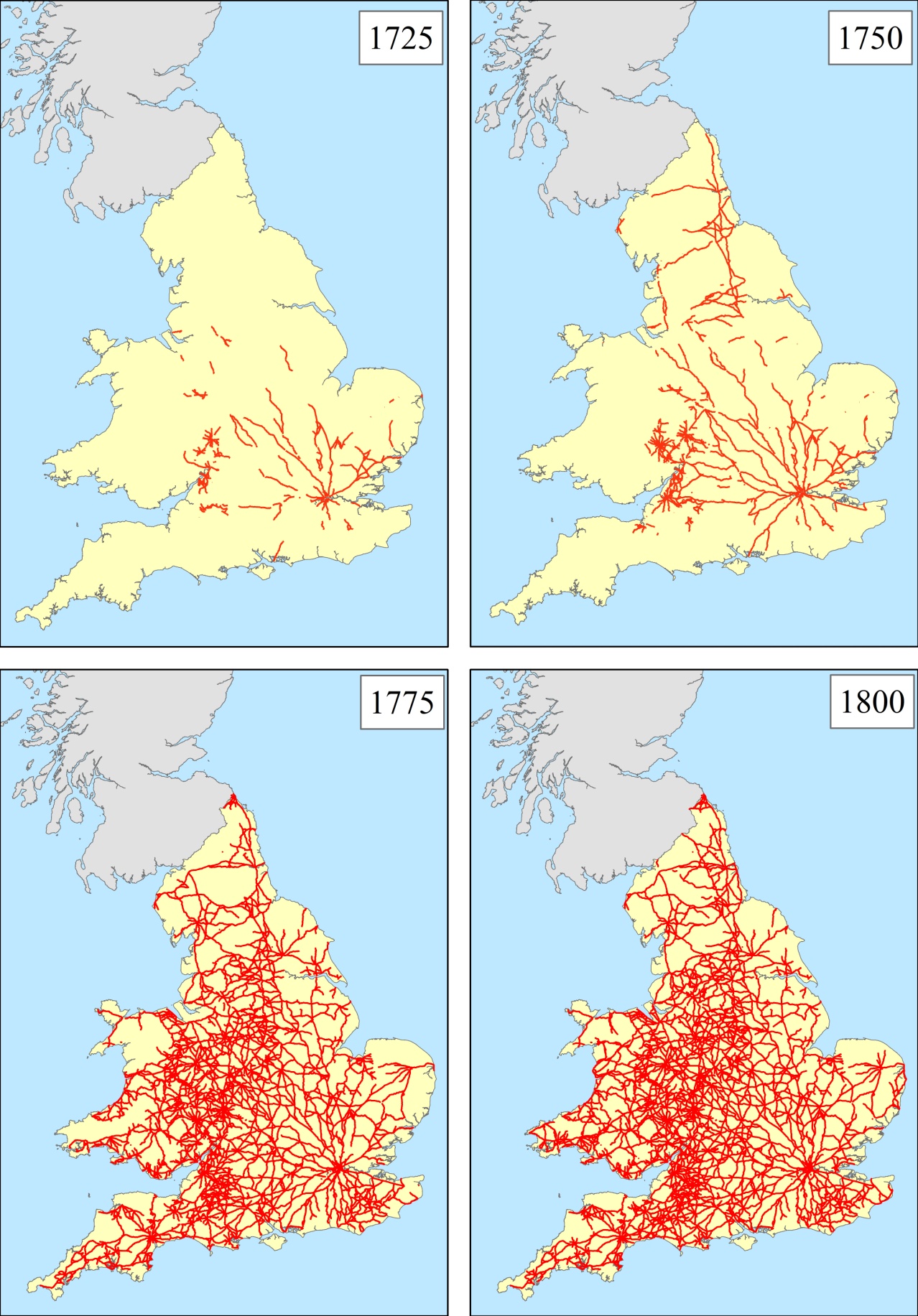


**Figure S2.** The turnpike road network, 1725-1800.

*Sources:* Cambridge Group for the History of Population and Social Structure GIS datasets (for a dynamic time series of the turnpike road network see <http://www.campop.geog.cam.ac.uk/research/projects/transport/data/turnpikeroadnetwork.html>).


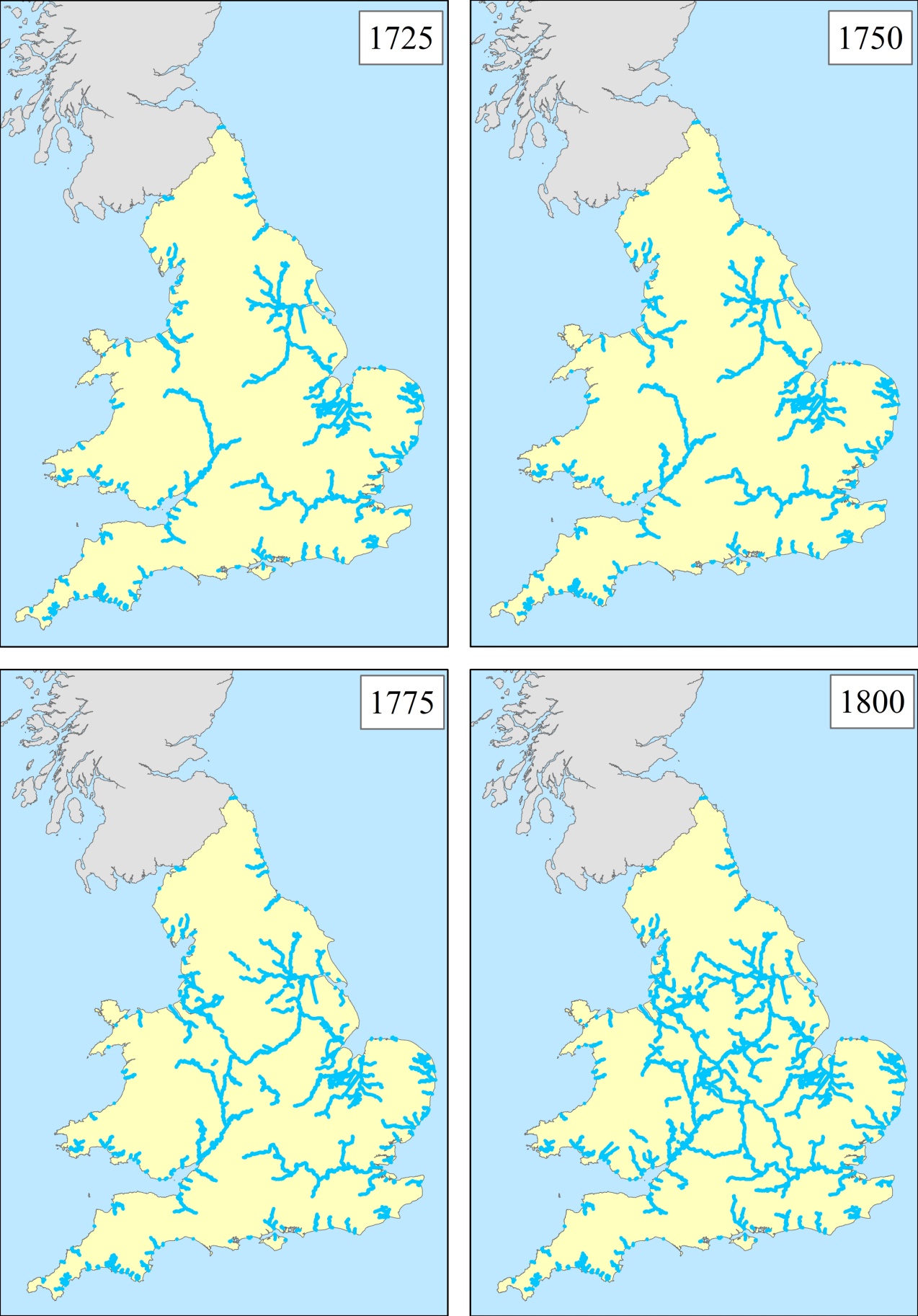


**Figure S3.** The navigable waterway network, 1725-1800.

*Sources:* Cambridge Group for the History of Population and Social Structure GIS datasets
